# Cross compatibility in intraspecific and interspecific hybridization in yam (*Dioscorea* spp.)

**DOI:** 10.1038/s41598-022-07484-x

**Published:** 2022-03-02

**Authors:** Jean M. Mondo, Paterne A. Agre, Alex Edemodu, Robert Asiedu, Malachy O. Akoroda, Asrat Asfaw

**Affiliations:** 1 International Institute of Tropical Agriculture (IITA), Ibadan, Nigeria; 2 Institute of Life and Earth Sciences, Pan African University, University of Ibadan, Ibadan, Nigeria; 3 Department of Crop Production, Université Evangélique en Afrique (UEA), Bukavu, Democratic Republic of the Congo; 4 Department of Agronomy, University of Ibadan, Ibadan, Nigeria

**Keywords:** Genetics, Plant sciences

## Abstract

Yam (*Dioscorea* spp.) is a staple crop for millions of people in the tropics and subtropics. Its genetic improvement through breeding is being challenged by pre-zygotic and post-zygotic cross-compatibility barriers within and among species. Studies dissecting hybridization barriers on yam for improving the crossability rates are limited. This study aimed to assess the cross-compatibility, which yielded fruit set, viable seeds and progeny plants in an extensive intraspecific and interspecific crossing combinations in a yam genetic improvement effort to understand the internal and exogenous factors influencing pollination success. Cross-compatability was analyzed at the individual genotype or family level using historical data from crossing blocks and seedling nurseries from 2010 to 2020 at the International Institute of Tropical Agriculture (IITA). The average crossability rate (ACR) was lower in interspecific crossing combinations (6.1%) than intraspecific ones (27.6%). The seed production efficiency (SPE) values were 1.1 and 9.3% for interspecific and intraspecific crosses, respectively. Weather conditions and pollinator's skills are the main contributors to the low success rate in the intraspecific cross combinations in yam breeding. At the same time, genetic distance and heterozygosity played little role. Interspecific cross barriers were both pre-zygotic and post-zygotic, resulting from the evolutionary divergence among the yam species. *Dioscorea rotundata* had higher interspecific cross-compatibility indices than *D. alata*. Distant parents produced intraspecific crossbred seeds with higher germination rates compared to closest parents (*r* = 0.21, *p* = 0.033). This work provided important insights into interspecific and intraspecific cross-compatibility in yam and suggested actions for improving hybridization practices in yam breeding programs.

## Introduction

Yam (*Dioscorea* spp.) is a crucial crop for food security and poverty alleviation in the tropics and subtropics^1^. Of the ~ 600 yam species, eleven are majorly produced for food and income^2^. Yam globally ranks fourth among the root and tuber crops and second after cassava in Africa^2,3^. In West Africa, where yam is extensively grown (> 90% global production), the per capita consumption can be as high as 40 kg per year for some ethnic groups^4^. In this region, the yam crop is an integral part of the socio-cultural and religious belief systems. Despite its importance as a staple food crop, source of income^5^, and socio-cultural connection to the society which depends on it, yam productivity has not increased much as compared to other root and tuber crops or cereal and grain crops^2,6^. Yam cultivation is challenged by many factors, of which an under-developed seed system is primary. In response to this significant challenge, there are breeding and seed system initiatives to improve the quality and availability of improved varieties and their seeds.

Yam breeding is instrumental in developing improved varieties, packaged in the form of seed, possessing superior yield and food quality attributes for different market segments and production environments. Breeding objectives are diverse and include high and stable tuber yield, outstanding nutritional and processing qualities, pest and disease resistance, and ecological adaptation traits^2^. To achieve these objectives, crop breeding relies on identifying desirable genes in source germplasm: landrace, breeding line, wild relatives, and their successful transfer to the agronomically and nutritionally preferred background. Success in this effort primarily depends on flowering, pollination, fruit and seed set, and seed germination^7,8^. However, yam breeding has been slow due to the alterations in reproductive ability that followed its domestication^9^. For instance, the domesticated yam species primarily propagate vegetatively via tubers or bulbils than the botanical seeds.

Consequently, flowering in most popular yam varieties became sparse, irregular, or absent. Besides, there are ploidy variations, asynchronous flowering between male and female individuals, high flower and ovule abortion rates, and low seed viability^7,9–12^. Cultivated yam is mainly dioecious, making many genotypes an obligate out-crosser. Natural cross-pollination is exclusively entomophilous, the sticky nature of pollen grains being unfavorable to wind pollen dispersal^7^. These factors result in low cross-pollination success causing low fruit and seed set from supervised and unsupervised crosses.

Broadening the existing yam breeding populations' genetic base is crucial for improving tuber yield, pest resistance, quality, and adaptation traits among cultivated yams. Landraces, secondary/tertiary gene pools, and wild relatives (primary gene pool) are unique sources of genes for these traits and thus should be exploited for yam breeding progress^13–16^. To date, intraspecific crosses have mainly been utilized to develop improved commercial yam varieties^2^. An attempt with an interspecific cross to introgress desired genetic variants among yam species has been reported^16^. However, such effort is often challenged by pre-zygotic and post-zygotic cross-compatibility barriers in most crops^17–19^, including the yams^20,21^. Although some yam species cross naturally in controlled and wild environments^22^, efforts are necessary to understand the nature of crossing barriers and devise means that facilitate gene flow among yam species to achieve cross-breeding goals. Most reports associated the low cross-compatibility among yam species with differences in ploidy status^7,16,20,21^. However, the causes and extent of interspecific barriers in yam have been poorly investigated and documented.

The International Institute of Tropical Agriculture (IITA) is a member of the Consultative Group for International Agricultural Research (CGIAR) with the global mandate for yam research. In partnership with its national and international partners, IITA maintains, develops, and releases yam varieties to meet farmers’ and other end-users needs and demands. However, past experiences in yam breeding revealed consistently low intra- and interspecific cross-compatibility rates throughout the years and stations. For instance, Darkwa et al.^2^ estimated the average crossability (ACR) and seed production efficiency (SPE) rates at 20.3 and 10.5%, respectively, for *D. rotundata* and 28.0 and 9.3%, respectively, for *D. alata* at the IITA, Ibadan, Nigeria. Although several reports referred superficially to the pollination success, cross-compatibility, and seed viability among and within yam species, very few studies entirely focused on these aspects. A retrospective analysis of the past pollination practices and information is crucial for a better understanding of factors linked to the reported low cross-compatibility rates in yam breeding and to suggest future directions. This study, therefore, explored the yam pollination information at the IITA for the period of 2010–2020 to specifically assess: (1) the cross-compatibility rates among and within yam species; (2) the germination rate of crossbred seeds, and (3) internal and external factors associated with yam cross-compatibility rates.

## Results

### Weather conditions at IITA breeding sites

At the Ibadan station, the temperature was stable across years while the rainfall was erratic and significantly fluctuated from year to year (Fig. S1a). The year 2010 had the highest rainfall (1926.3 mm) while 2020 had the lowest (1074.0 mm). June (246 mm, 15.5 days) and September (245 mm, 17.5 days) were the wettest months of the year. During the yam flowering window, August (130 mm, 13.9 days) and November (31.9 mm, 3.3 days) were the driest months. The mean minimum and maximum temperatures were 22.4 and 31.6 °C, respectively. Ibadan received a mean annual rainfall of 1546.9 mm from 116 rainy days, mean sunshine of 5.8 h, and mean maximum and minimum relative humidity of 92.8 and 50.25%, respectively (Fig. S1b). As for Ibadan, Abuja station had stable temperatures across years (min. 21.12 – 21.77 °C, max. 32.21 – 33.48 °C) while there was high inter-annual rainfall variability (945 mm in 2015 – 2105.88 mm in 2019) (Fig. S1c). At Abuja, the flowering window had well distributed monthly rainfall amounts (217 mm, 20 rainy days from June-October) with moderate temperatures (21.92 °C min, 29.93 °C max). August was the wettest month (276 mm, 25 rainy days), while November was the driest (11 mm, 5 days) within the yam flowering window. Abuja minimum and maximum relative humidity values were 48.84 and 90.97%, respectively. This station experienced a wind speed of 1.89 km h^−1^ and solar radiation of 14.94 MJ m^−2^ day^−1^ (Fig. S1d). The two stations were significantly different for maximum temperatures (t = − 4.62, *p* = 0.006), minimum temperatures (t = 6.54, *p* = 0.001), and wind speed (t = 7.90, *p* < 0.001). All other parameters showed no differences between the two stations.

### Cross-compatibility within and among yam species

Between 2010 and 2020, the IITA yam breeding program made ~ 332,500 and 176,645 hand pollinations in *D. rotundata* and *D. alata* crossing blocks, respectively (Table 1). Other yam species were seldom used in intra-specific hybridizations. The average cross-compatibility rates (ACR) among *D. rotundata* and *D. alata* genotypes were 23.4 and 31.7%, respectively. *Dioscorea rotundata* (9.2%) and *D. alata* (9.3%) had comparable seed production efficiency (SPE) values.

**Table 1:** Intraspecific cross-compatibility indices within *D. alata* and *D. rotundata* genotypes.

Species	Females	Males	Cross-combinations	Yearly crosses	Flowers pollinated	ACR (%)	SPE (%)
*D. alata*	205	229	796	16,058.64 ± 8144.28	176,645	31.73 ± 18.89	9.31 ± 6.50
*D. rotundata*	365	347	1266	30,228.09 ± 13,113.77	332,509	23.40 ± 18.63	9.16 ± 3.76
Sum/mean (± SD)	570	576	2762	23,143.37 ± 10,629.02	509,154	27.57 ± 18.76	9.23 ± 5.13

ACR = average crossability rate, SPE = seed production efficiency. SD = standard deviation. Yearly crosses, ACR and SPE are presented by means ± standard deviation.

The highest interspecific ACR were recorded on *D. rotundata* when crossed with its wild relatives, *D. burkiliana* (29.2%) and *D. hirtiflora* (27.3%). Interspecific crosses involving *D. alata* had consistently lower ACR (Table 2). The highest interspecific SPE was recorded on *D. rotundata* × *D. burkiliana* (9.7%).

**Table 2:** Interspecific cross-compatibility indices among yam species (*Dioscorea* spp.)

Species and crosses	Females	Males	Cross-combinations	Flowers pollinated	ACR (%)	SPE (%)
*D. alata* × *D. bulbifera*	9	1	9	5710	3.66 ± 12.40	0.51 ± 7.31
*D. alata* × *D. bulbifera* wild	4	4	7	484	2.04 ± 7.41	0.00 ± 0.00
*D. alata* × *D. cayenensis*	4	3	6	927	0.00 ± 0.00	0.00 ± 0.00
*D. alata* × *D. dumetorum*	4	6	7	337	0.00 ± 0.00	0.00 ± 0.00
*D. alata* × *D. rotundata*	8	23	28	2807	5.31 ± 17.18	3.12 ± 14.57
*D. bulbifera* wild × *D. rotundata*	2	4	5	520	1.21 ± 2.46	0.07 ± 0.22
*D. bulbifera* × *D. alata*	14	5	21	2795	0.44 ± 2.11	0.00 ± 0.00
*D. bulbifera* × *D. rotundata*	9	7	19	3773	1.16 ± 6.28	0.09 ± 0.64
*D. dumetorum* × *D. alata*	4	2	6	181	11.80 ± 16.57	0.00 ± 0.00
*D. dumetorum* × *D. rotundata*	3	1	3	299	10.59 ± 18.16	0.00 ± 0.00
*D. hirtiflora* × *D. rotundata*	1	3	3	466	27.33 ± 35.80	0.52 ± 2.18
*D. praehensilis* × *D. alata*	3	3	6	2146	3.66 ± 3.42	-
*D. praehensilis* × *D. rotundata*	3	6	8	2400	11.97 ± 12.13	5.30 ± 7.14
*D. rotundata* × *D. alata*	7	5	12	4626	0.44 ± 2.52	0.09 ± 0.78
*D. rotundata* × *D. bulbifera*	8	4	11	6770	0.59 ± 3.72	0.24 ± 1.65
*D. rotundata* × *D. bulbifera* wild	7	1	7	5769	1.60 ± 7.31	0.38 ± 2.90
*D. rotundata* × *D. burkilliana*	3	1	3	680	29.20 ± 24.59	9.67 ± 9.21
*D. rotundata* × *D. cayenensis*	21	11	58	26,813	6.46 ± 13.24	0.77 ± 3.92
*D. rotundata* × *D. dumetorum*	3	1	3	221	0.00 ± 0.00	0.00 ± 0.00
*D. rotundata* × *D. hirtiflora*	6	1	6	1278	0.67 ± 3.11	0.00 ± 0.00
*D. rotundata* × *D. praehensilis*	1	1	1	102	9.80 ± 13.00	-
Sum/mean (± SD)	118	91	229	69,104	6.09 ± 9.59	1.09 ± 2.66

The first species in the pedigree served as the female parent in the interspecific cross while the second species provided the male parents, ACR = average cross-compatibility/crossability rate, SPE = seed production efficiency. *Dioscorea bulbifera* wild refers to individuals of the species from the forest/wild environments. SD = standard deviation. ACR and SPE are presented by means ± standard deviation.

### Exogenous and genetic factors on yam cross-pollination success

#### Exogenous factors

Year, location, and pollinator effects significantly influenced the supervised cross-pollination success in yam (Tables 3, 4, 5, Table S5). The cross-pollination success indices such as the ACR and SPE were consistently higher at the Ibadan site than the Abuja on both *D. rotundata* and *D. alata* crossing blocks (Table 4). The highest ACR values, 27.7% on *D. rotundata* and 69.4% on *D. alata* crossing blocks were recorded in 2019. The lowest 10.7% on *D. rotundata* and 12.3% on *D. alata* crossing blocks were in 2017 and 2020, respectively (Table 5).

**Table 3:** Summary ANOVA of the influence of year, location, and cross-combinations on pollination success at IITA (2010–2020).

Sources of variation	ACR (%)		SPE (%)	
	MS	*p *value	MS	*p *value
***D. rotundata***				
Year	4521.9	< 0.001	1184.87	< 0.001
Location	12,027.0	< 0.001	935.39	< 0.001
Cross combination	399,876	< 0.001	116.88	< 0.001
***D. alata***				
Year	31,586	< 0.001	5476.8	< 0.001
Location	36,431	< 0.001	1897.8	< 0.001
Cross combination	460	< 0.001	336.9	< 0.001

*ACR* = *average cross-compatibility/crossability rate, SPE* = *seed production efficiency, MS* = *mean squares.*

**Table 4:** Pollination success across IITA breeding sites (Ibadan vs. Abuja) for *D. alata* and *D. rotundata.*

Species	Abuja		Ibadan	
	ACR (%)	SPE (%)	ACR (%)	SPE (%)
*D. rotundata*	14.05 ± 14.61	5.77 ± 7.55	24.40 ± 21.05	9.58 ± 11.45
*D. alata*	9.84 ± 12.40	2.95 ± 5.38	38.04 ± 27.53	11.85 ± 13.11

ACR = average crossability rate, SPE = seed production efficiency. ACR and SPE are presented by means ± standard deviation.

**Table 5:** *D. rotundata* and *D. alata* average crossability rate (ACR) and seed production efficiency (SPE) for 2010–2020 at IITA.

Year	Female	Male	Cross combination	Flowers pollinated	ACR (%)	SPE (%)
***D. rotundata***						
2010	8	7	14	1831	22.63 ± 19.17	9.00 ± 8.53
2011	5	6	16	5495	14.73 ± 15.19	6.30 ± 7.10
2012	23	13	102	39,891	21.49 ± 19.94	-
2013	21	28	101	22,619	23.27 ± 18.32	6.41 ± 8.69
2014	22	18	93	26,019	24.10 ± 18.31	8.64 ± 8.88
2015	20	21	73	23,780	14.16 ± 14.46	7.23 ± 6.91
2016	62	45	168	65,668	19.02 ± 17.06	7.14 ± 7.57
2017	31	32	125	47,864	10.70 ± 12.12	2.32 ± 3.51
2018	24	16	77	22,471	16.18 ± 14.30	6.90 ± 7.32
2019	72	73	236	36,371	27.67 ± 23.05	11.35 ± 13.47
2020	77	88	261	40,397	27.26 ± 23.23	11.82 ± 13.19
Sum/mean (± SD)	365	347	1266	332,509	23.40 ± 18.63	9.16 ± 3.76
***D. alata***						
2010	6	8	13	2251	26.08 ± 13.72	8.86 ± 6.64
2011	10	8	34	7187	23.21 ± 19.14	5.90 ± 4.98
2013	32	28	115	26,600	30.90 ± 22.05	9.08 ± 11.32
2014	22	12	74	22,144	41.47 ± 18.02	11.41 ± 8.26
2015	14	11	50	26,199	26.52 ± 21.13	4.00 ± 6.53
2016	35	59	169	38,348	22.99 ± 19.58	6.49 ± 6.83
2017	27	20	117	24,189	32.54 ± 20.83	9.35 ± 6.89
2019	42	62	158	17,029	69.45 ± 23.24	25.54 ± 14.56
2020	17	21	66	12,698	12.28 ± 21.53	3.32 ± 10.12
Sum/mean (± SD)	205	229	796	176,645	31.73 ± 18.89	9.31 ± 6.50

ACR = average crossability rate, SPE = seed production efficiency. ACR and SPE are presented by means ± standard deviation (SD).

**Figure 1:**
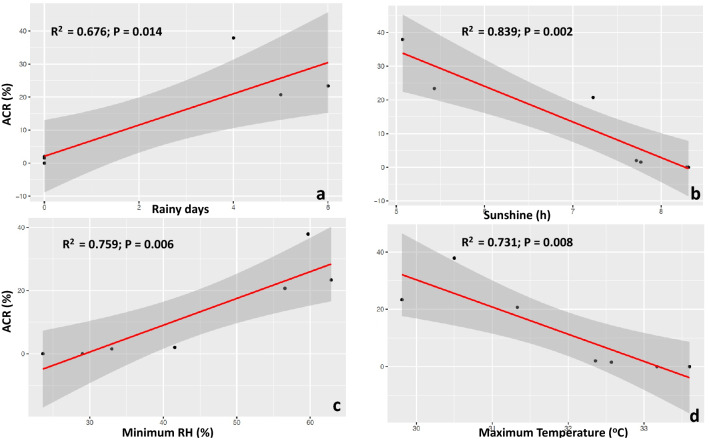
Weather parameters bearing influence on *D. alata* crossability rate (ACR) at IITA, Nigeria: **(a)** number of rainy days, **(b)** sunshine, **(c)** minimum relative humidity (RH), **(d)** maximum temperature.

**Figure 2:**
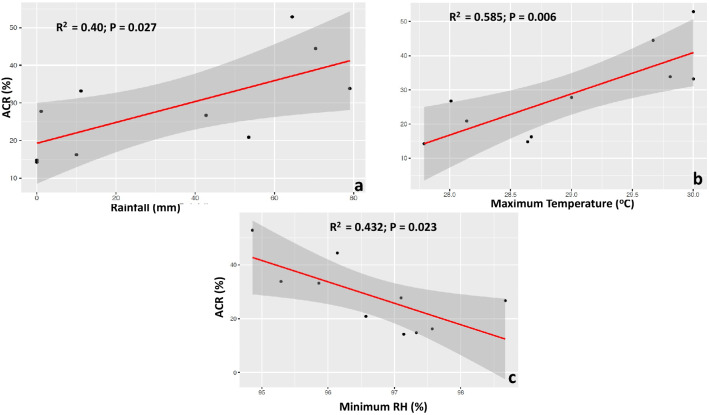
Weather parameters bearing influence on *D. rotundata* crossability rate (ACR) at IITA, Nigeria: **(a)** rainfall, **(b)** maximum temperature, **(c)** minimum relative humidity (RH).

**Figure 3:**
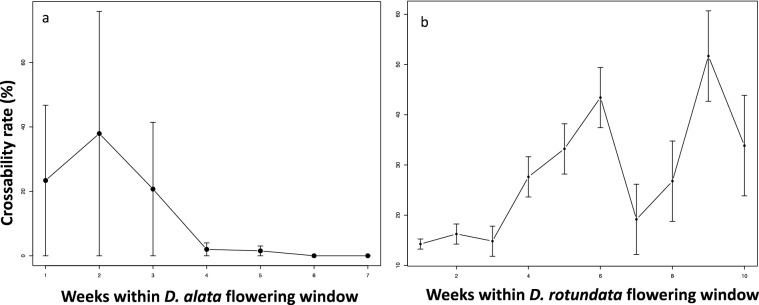
Pollination success along yam flowering window: **(a)** *D. alata* and **(b)** *D. rotundata.* Week 1 for *D. alata* corresponded to the second week of October while week 7 was the fourth week of November. Week 1 for *D. rotundata* referred to first week of August while week 10 corresponded to the second week of October.

The annual weather data did not fully explain the inter-annual variability in yam cross success. Instead, weekly variability in rainfall, temperature, relative humidity, sunshine, and the number of rainy days within the yam flowering window (July to November) significantly influenced the pollination outcomes in either the *D. rotundata* or *D. alata* crossing blocks (Figs. 1 and 2, Table S6). In August, a lower success rate was recorded in the *D. rotundata* crossing block, while this happened in the *D. alata* crossing block in November. Crosses made after 14 November had no fruit setting regardless of the cross-combinations in *D. alata* (Fig. 3).

Technicians involved in yam hand pollination also played a significant role in the cross-pollination success rates (Table S5). Some technicians achieved up to 5 times more pollination success rates than others. When assessed on the same sets of parents and cross-combinations, the pollinator's efficiency ranged from 6.2–30.3% and 27.9–67.4% in *D. alata* and *D. rotundata* pollination blocks, respectively.

#### Genetic relatedness and cross-pollination success

**Figure 4:**
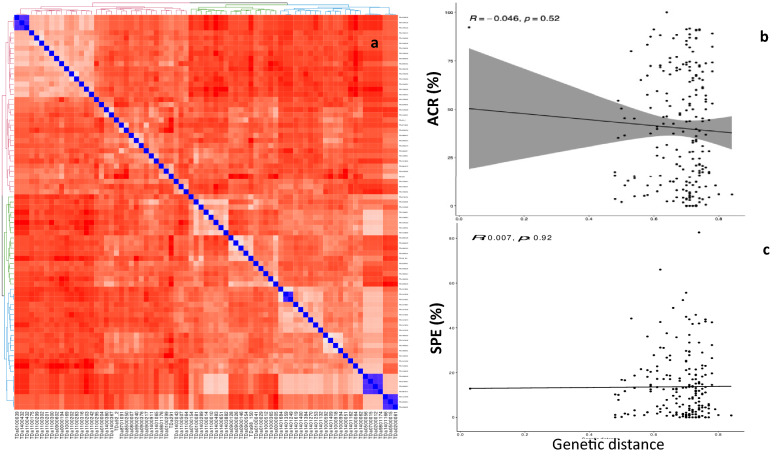
Genetic relatedness among *D. alata* genotypes using SNP information: **(a)** Heatmap IBS genetic distance, and influence on **(b)** average crossability rate (ACR) and **(c)** seed production efficiency (SPE) in *D. alata* cross-combinations.

Genetic distance among *D. alata* parental clones ranged from 0.02 to 0.84 (Fig. 4a, Supplementary data 1). There were no significant correlations between the parental genetic distance and the ACR (*r* = 0.046, *p* = 0.52) and between the parental genetic distance and the SPE (*r* = 0.007, *p* = 0.92)(Fig. 4b and 4c).

**Figure 5:**
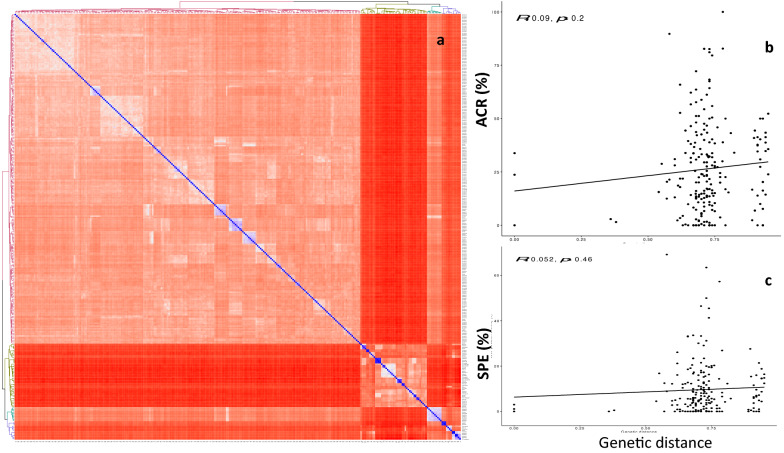
Genetic relatedness among *D. rotundata* genotypes using SNP information: **(a)** Heatmap IBS genetic distance, and influence on **(b)** the average crossability rate (ACR) and **(c)** seed production efficiency (SPE) in *D. rotundata* cross-combinations.

The *D. rotundata* clones used in crosses had a genetic distance of 0.01–0.96 (Fig. 5a, Supplementary data 2). No relationship was established between parental genetic distances and the ACR (*r* = 0.09, *p* = 0.2) or the parental genetic distance and the SPE (*r* = 0.052, *p* = 0.46) for *D. rotundata* cross-combinations (Fig. 5b and 5c**)**.

#### Heterozygosity and cross-compatibility

**Figure 6:**
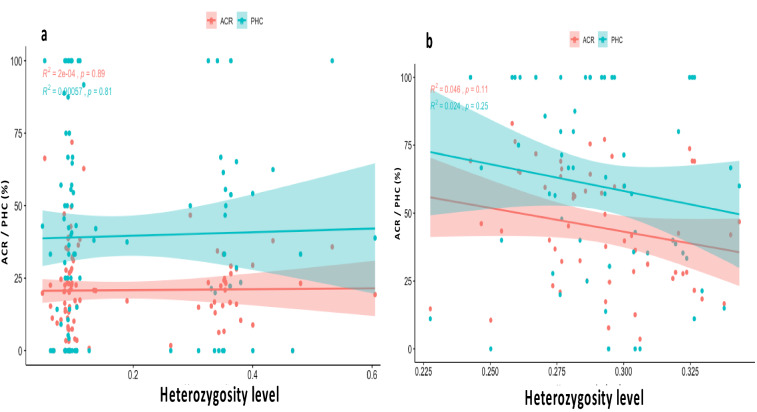
Heterozygosity level of **(a)** *D. rotundata* and **(b)** *D. alata* parental genotypes, and influence on the average crossability rate (ACR) and the percentage high crossability (PHC).

The heterozygosity level of the parents had no significant effect on the ACR of the *D. rotundata* (*r* = 0.014, *p* = 0.89, Fig. 6a) and *D. alata* genotypes ( *r* = 0.214, *p* = 0.11, Fig. 6b). The same trend was observed for the genotype’s percentage high crossability (PHC) irrespective of the species (*r* = 0.024, *p* = 0.81 for *D. rotundata* and *r* = 0.155, *p* = 0.25 for *D. alata*).

Although no significant effect of genetic relatedness and heterozygosity of crossed parents was established on cross-compatility indices, there was an influence (*p* < 0.001) of parent combinations on ACR and SPE for both major yam species (Table 3).

### Seed germination rates of major yam species

**Figure 7:**
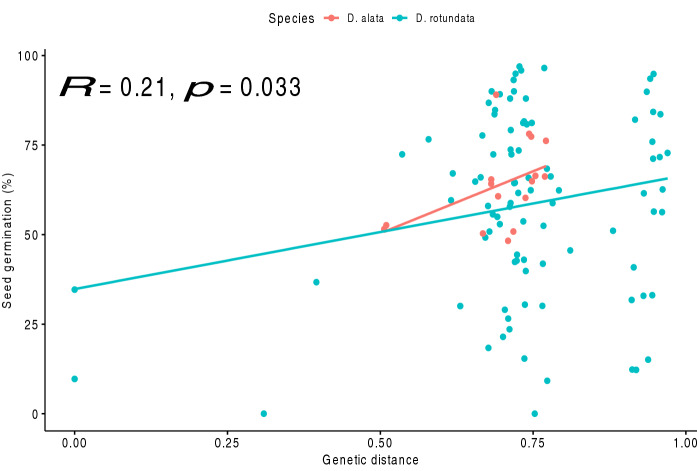
Correlation between the parents’ genetic distance and seed germination rates in *Dioscorea* spp.

Germination rates significantly varied among years (*p* = 0.0003) for *D. alata* and *D. rotundata* crossbred seeds. The highest seed germination rate was recorded in 2020 (54%), while the lowest rate, 37%, was in 2015. The seed germination rate was significantly different among the supervised interspecific and intraspecific crossbred seeds and the unsupervised open-pollinated intraspecific crosses (*p* < 0.001) (Table S7). The open-pollinated seeds of *D. rotundata* germinated most (61%), while that of *D. alata* were least viable (43%). However, intraspecific *D. alata* cross-bred seeds (55%) outperformed the *D. rotundata* intraspecific cross-bred seeds (49%). We noticed a positive correlation (*r* = 0.21, *p* = 0.033) between the seed germination rates and the parents’ genetic distance (Fig. 7). Seed germination rates of major *D. alata* and *D. rotundata* intraspecific cross-combinations are presented in Table S8.

## Discussion

This study used 11-year historical data to investigate intra- and interspecific cross-compatibility and seed germination rates in yam. We ascertained the generally low cross-compatibility rates among and within yam species reported by several studies^2,7,12,16,23–26^. Our retrospective analysis in yam crossing blocks at the IITA breeding program indicated that climatic factors and technician pollination skill are the main exogenous factors contributing to low cross-pollination success rates in *D. alata* and *D. rotundata.*

Among the climatic factors, weekly differences in rainfall, temperature, relative humidity, number of rainy days, and sunshine during the pollination window were the main contributors to success rates. Our results revealed that making crosses in August for *D. rotundata* and November for *D. alata* could result in low pollination success due to the suboptimal weather conditions. This suggests the need for supplemental irrigation in yam crossing blocks to reduce the water deficit's adverse effects on yam reproductive phases during these months. Our results supported those of Abraham and Nair^27^, which linked successful pollination in yam with high relative humidity and moderate atmospheric temperatures.

Examining technicians' performance on the same cross-combinations simultaneously, we found highly significant differences among technicians in making successful pollination (*p* < 0.001). The anther removal from the male flower and its transfer to the female stigma is delicate. It requires special skills that might be lacking in many short-term pollinator staff involved in yam hand pollination operations. Pollinator's skill and weather conditions partly explained the significantly lower pollination success recorded at the IITA Abuja site than at Ibadan. Abuja site was characterized by lower relative humidity and higher temperatures compared to Ibadan. Hence, selecting conducive crossing sites and enhancing technicians' skills are highly relevant to improve the cross-pollination success rates in yam breeding programs.

Genotype genetic relatedness had no significant effect on pollination success rates in both *D. rotundata* and *D. alata* intraspecific crosses. The high or low success rates were recorded in crosses irrespective of the genetic distance between parents. This wide genetic compatibility range implies that breeding programs could exploit desirable genetic variant gene transfer within closely related and distant parents within the yam species. Although admitting that inbreeding depression could occur with low genetic distances, Willi and Van Buskirk^28^ found no single peak of optimal genetic outbreeding in outcrossing plants. Since the genetic distance had no significant effect on yam cross-compatibility rates, it might be wise to cross genetically distant parents to exploit heterosis in yam breeding. Past studies supported that tuber yield is positively correlated with heterozygosity in yam^16,20,21^. It is, however, noteworthy that outbreeding over more considerable genetic distances could lead to a decline in the clonal component of fitness, as warned by Willi and Van Buskirk^28^.

Although no significant effect of genetic relatedness and heterozygosity of crossed parents was established on cross-compatility indices, there was an influence of parent combinations on ACR and SPE for both major yam species. Using the same *D. alata* plant materials as in this study, genome-wide association studies (GWAS) showed that ACR could be controlled by loci on chromosomes 1, 6 and 17^12^. That study, therefore, opened an avenue for developing genomic tools for predicting hybridization success in yam breeding programs.

As previously suggested by Mondo et al.^7^, several other internal and exogenous factors seem to influence yam pollination successes and should be given attention in future studies. Factors such as the optimum pollination time, floral morphology, and biology, especially for anthesis detection, pollen parent viability, etc., should also be investigated to identify additional causes of low intraspecific pollination success in yam at IITA. To complement this study conclusions, we recommend a more refined experiment on ploidy status among and within species to elucidate its effects on intra and interspecific cross-pollination success.

Pre-breeding involving interspecific hybridizations is valuable for broadening the cultivated yam species' genetic base by introducing desirable genes. Past efforts of broadening the genetic bases of white (*D. rotundata*) and water (*D. alata*) yams involved six relative species at IITA. Those relative yam species served as valuable sources of genes for yam mosaic virus (*D. praehensilis*), bulbil formation ability as alternative planting materials (*D. bulbifera*), source of carotenoids (*D. cayenensis*), tolerance to nematodes (*D. dumetorum*) or as bridge between incompatible species (*burkilliana* and *D. hirtiflora*) (Table S9). Although some natural/spontaneous interspecific hybridizations between *D. rotundata* and its wild relatives has been reported in West Africa^22,29^, yam is only propaged using tubers by farmers and botanical seed is only used for breeding purposes. Spontaneous crosses would require parental reconstruction to identify the pollen parent before advancing progenies. Developing bi-parental crosses through hand pollination is thus more precise. Average crossability rates, through hand pollination, were highest when crossing *D. rotundata* with its wild relatives (*D. burkilliana* and *D. hirtiflora*). The same trend was observed on SPE as the highest values were on *D. rotundata* × *D. burkilliana* cross-combinations. This relatively higher cross-compatibility rate could be attributed to high genetic relatedness among these species, as reported by previous studies^22,25,29–31^. However, interspecific crosses involving *D. rotundata* with *D. cayenensis* had generally low success rates due to expected differences in ploidy level. *Diocorea cayenensis* is mostly triploid with male sex flowers, while diploid plants dominate *D. rotundata* with dioecious flowering patterns^32,33^. It is noteworthy that successful interspecific crosses were dependent on the cross direction such that reciprocals seldom ensured satisfactory results. This demonstrated the presence of unilateral incompatibility in yam species translated by rejection, in one crossing direction, of pollen from one species by pistils of a related species^19,34^. Therefore, when designing an interspecific hybridization program, male and female parents should be selected carefully to optimize the pollination success rates.

**Figure 8:**
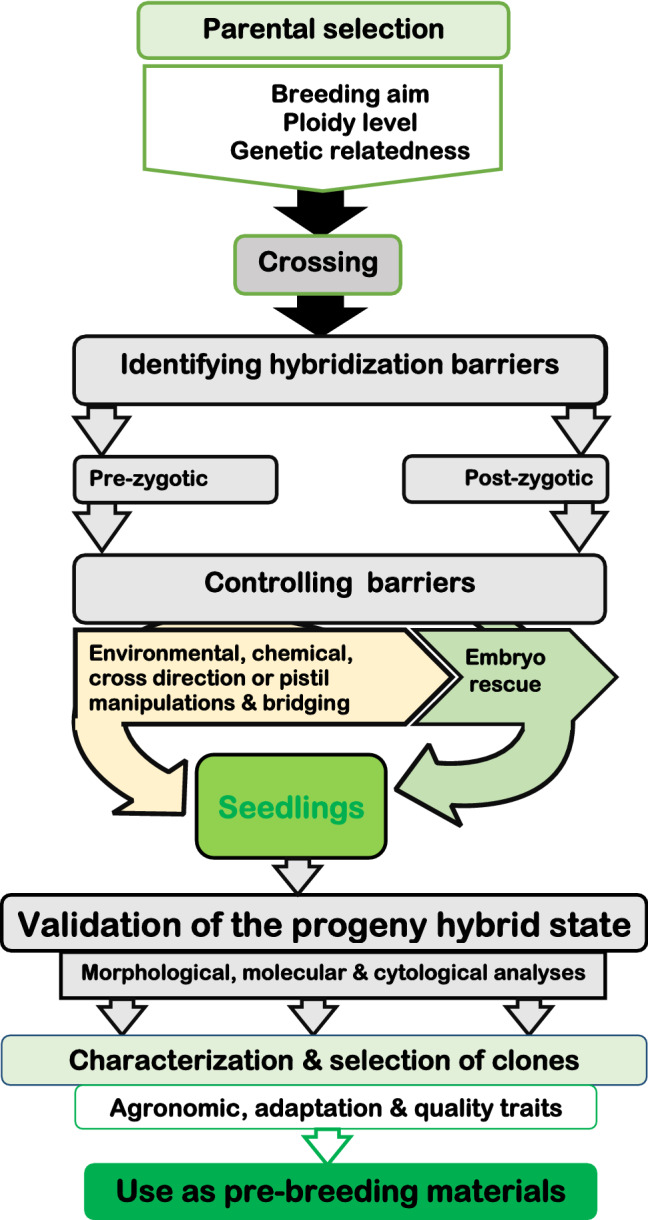
Scheme for creating a viable interspecific breeding program for yam.

Our results showed that yam species experience both pre‐zygotic and post-zygotic compatibility barriers. The pre-zygotic barriers prevented successful fertilization even when viable pollen and receptive stigmatic parents were used. The pre‐zygotic barriers seemed much stronger among yam species as the crossability rate (translated by fruit set) was rare in interspecific crosses. Luque et al.^19^ associated such barriers to the degree of domestication. Besides, that pre-zygotic barrier, also referred to as pre-fertilization barrier, could be a result of the halfway arrest of the elongation of the pollen tube in the stylar canals^18^. On the other hand, post-zygotic barriers prevented embryo development and seed formation even though the fertilization was successful. Such post-zygotic barriers could be mainly attributed to differences in ploidy levels^19^ or by degradation of hybrid embryos and/or endosperm in incongruous crossings^18^. For successful interspecific yam hybridizations, both barriers need to be overcome. Direct in vitro pollination is among the techniques for overcoming the pre-zygotic incompatibility barriers in yams^7^. Besides, hybrid genotypes acting as a bridge between related incompatible yam species could be developed^19^ or to experiment the cut-style pollination technique which had been successful in other crops^18^. The post-zygotic barriers resulting in seedless fruits can be controlled by biotechnology techniques such as the embryo rescue to prevent post-zygotic abortions^17^. Such techniques have been fully developed and implemented by CIRAD-Guadeloupe, France, and could be transferred to other programs to exploit interspecific and interploidy crosses in yam breeding^20,21^. We have suggested a scheme illustrating how a successful interspecific hybridization can be designed in yam breeding programs (Fig. 8).

*Dioscorea rotundata* had higher seed germination under unsupervised open pollination than controlled hand pollination. Pollen donors of higher quality might have been involved in open pollination than those used in hand pollination^35^. This study showed also that distant parents produced highly viable crossbred seeds than closely related ones. This could be associated with the hybrid vigor of distant parents’ progenies. Fischer et al.^35^ argued that low genetic distance among crossed individuals reduces cross-compatibility and offspring fitness in species with a self-incompatibility system. This reduced offspring fitness could have translated into low seed germination and poor seedling vigor due to inbreeding depression. We also found seed germination rate improvement across years as a result of growth medium optimization at IITA since 2014. Most seedling nurseries have been using carbonized rice husk under proper management in screenhouse conditions^36^. Therefore, the seed germination rate is not only under genetic control but also influenced by growing media and conditions.

## Conclusions

This study ascertained the generally-reported low crossability and seed setting rates among and within yam species. Low success rates in yam crossing blocks were mainly attributed to the suboptimal weather conditions and technicians’ skill rather than the genetic relatedness and parents’ heterozygosity levels, as previously assumed. However, the seed germination rate was highest for crossbred seeds from genetically distant parents. Many other factors seem to influence the yam pollination success and should be investigated in future studies.

## Materials and methods

### Plant materials and weather conditions

In this study, 11-year (2010–2020) historical data generated from the IITA Yam Breeding Unit’s crossing blocks and seedling nurseries were explored. Information on intra- and interspecific cross-compatibility and seed germination rates was gathered irrespective of the breeding objectives. Eight yam species, sourced from IITA core collection, were used for inter- and intraspecific cross-combinations. These species are from different sections of the genus *Dioscorea*. *Dioscorea alata* L., *D. rotundata* Poir., *D. cayenensis* Lam., *D. burkilliana* J. Miège, and *D. praehensilis* Benth. belong to the *Enantiophyllum* section. *Dioscorea dumetorum* (Kunth) Pax belongs to section *Lasiophyton*, while *D. bulbifera* L. and *D. hirtiflora* Benth are from the sections *Opsophyton* and *Asterotricha*, respectively. It is noteworthy that much of the yam breeding in IITA focused on the two most important cultivated species, *D. rotundata* and *D. alata,* with ~ 500,000 crosses recorded from 2010–2020. The list of most popular male and female *D. rotundata* and *D. alata* genotypes, their ploidy level, the type, the trait of interest, and average cross-compatibility rate information are provided in Table S1-4. The IITA yam crossing blocks were established at Ibadan (7°29′ N and 3°54′ E) and Abuja (9°10′ N and 7°21′ E) stations in Nigeria. Daily weather data of 2010–2020 were obtained from the IITA Geographic Information System Unit and summarized using a cross-tabulation function. At both IITA stations, yam fields were established in April. The flowering window extended from the end of June to early October for *D. rotundata* and the end of September to November for *D. alata*. Annual and monthly weather data are summarized in Fig. S1.

### Cross-compatibility analysis

Historical data on the number of flowers pollinated, and the corresponding fruit and seed sets for the different cross-combinations conducted in IITA yam crossing blocks from 2010 to 2020 were used to calculate the cross-compatibility indices: ACR, PHC, and SPE. The cross-compatibility rate of a cross was calculated using the following formula:

1 $$Crossability\, rate \left(\%\right)=\frac{Number\, of\, fruits\, set}{Number\, of\, flowers\, pollinated} \times 100$$

The ACR for a parent was calculated as the sum of cross-compatibility rates in specific crosses divided by the number of cross-combinations involving that particular parent:

2 $$ACR=\frac{\sum \,Crossability\, rates}{Number \,of\, cross\, combinations}$$

The PHC for a parent was calculated as the number of times the cross-compatibility rate exceeded the species overall cross-compatibility divided by the number of cross-combinations in which that parental genotype was involved:

3 $$PHC \left(\%\right)=\frac{Number\, of\, crossability\, rates >\,overall \,mean}{Number\, of\, cross\, combinations} \times 100$$

The SPE for a cross was calculated as the number of viable seeds divided by six times (the expected number of seeds in a yam fruit is six) the number of pollinated flowers multiplied by 100:

4 $$SPE \left(\%\right)=\frac{Number\, of\, viable\, seeds \,set}{Number\, of\, flowers \,pollinated \times 6}\times 100$$

### Analysis of genetic relatedness and heterozygosity level

To assess the influence of the genetic distance between crossed parents on ACR and SPE, Jaccard pairwise dissimilarity matrices were generated using philentropy package implemented in R^37^. This genetic relatedness analysis was performed using single nucleotide polymorphism (SNP) markers. This analysis involved 77 *D. alata* genotypes previously sequenced using Diversity Array Technology (DArT-Seq)^12,38^. SNP information of 302 *D. rotundata* genotypes was extracted from the genotyping by sequencing (GBS) studies reported in Bhattacharjee et al.^39^. The heterozygosity level of the different parental genotypes was then estimated using Tassel 5^40^. It is noteworthy that analyses on genetic relatedness and heterozygosity were only performed on the two economically-important yam species, *D. alata* and *D. rotundata*, for which sufficient molecular information was available. Unlike the estimation of cross-compatibility indices which included historical crossing data of all the genotypes (434 *D. alata* and 712 *D. rotundata*) maintained in the yam breeding at IITA, analyses of genetic relatedness and heterozygosity level only focused on popular clones (77 *D. alata* and 302 *D. rotundata*) for which both sequencing and crossing information were available.

### Crossbred seed germination rate assessment

The crossbred seed germination rates were determined using sample data of 2014–2020. The seed germination rate was estimated by dividing the seedling stand count in nurseries by the number of seeds sown multiplied by 100:

5 $$Seed \,germination \,rate (\%)=\frac{Number\, of\, seeds\, germinated }{Number\, of\, seeds \,sown}\times 100$$

Seed germination rate was further associated with parents’ genetic distance information to assess the cause-effect relationship.

### Determination of weather parameters and pollinator effects on pollination success

We used ten weather parameters to assess their influence on the cross-pollination success in yam crossing blocks. The weather parameters assessed were rainfall (mm), evaporation (mm), wind speed (km h^−1^), solar radiation (MJ m^−2^ day^−1^), minimum and maximum temperatures (°C), minimum and maximum relative humidity (%), sunshine (h) and the number of rainy days (a day was considered rainy when rainfall amount exceeded 0.5 mm). The weather parameters records on a yearly, monthly, weekly, and daily basis were assessed using multiple regression analysis. Differences in meteorological parameters at the two crossing sites, Ibadan and Abuja, were assessed using a t-test after testing the data’s normal distribution using the Shapiro–Wilk test.

The pollinator effect was assessed on selected 2020 *D. alata* and *D. rotundata* crossing blocks at the Ibadan station using the same cross-combinations, crossing time, and same technicians. A total of eight technicians were monitored per yam species (a separate record was filled for each pollinator without their awereness and data was analysed anonymously). It is noteworthy that the two yam species’ flowering windows are spaced in time, June to early October for *D. rotundata* and the end of September to November for *D. alata.* Therefore, a different set of pollinators was used for each species since crossing activity is mostly performed by short-time staff.

### Data analysis

Historical data on ACR, SPE, and seed germination rate were summarized by means and standard deviations. Pearson’s correlation was estimated to assess the relationship between ACR and SPE with internal (genetic relatedness, heterozygosity level) and external (weather conditions) factors. Effects of the year, location, and cross-combination on cross-compatibility indices were determined using the analysis of variance (ANOVA). ANOVA was also used to assess differences in seed germination rates among crossbred seeds from interspecific, intraspecific and open-pollinations. Means were further separated by Fisher’s least significant difference (LSD) test at 5% *p*-value threshold. The relationship of parents’ genetic distance with crossbred seed germination rate was also assessed using Pearson’s correlation analysis. Heatmap function implemented in package ‘qgraph’^41^ was used to estimate the genetic relationship among crossed parents. Results were presented as a heatmap with different color gradients based on genetic distance. Regression plots were then drawn to determine the level of significance and influence of genetic distance on ACR, SPE, and seed germination rate. Regression analysis was also used to assess the impact of parents' heterozygosity level on the ACR and PHC values. Pollinating technician’s effect on yam pollination success was evaluated using ANOVA and Tukey HSD All-Pairwise Comparisons Test at 5% *p*-value threshold.

## Supplementary Information

Supplementary Information 1.

Supplementary Information 2.

Supplementary Information 3.

## Data Availability

The original contributions presented in the study are included in the article/Supplementary Material. Further inquiries can be directed to the corresponding author.
