# Supplementary material for: Cross compatibility in intraspecific and interspecific hybridization in yam (Dioscorea spp.)
Source: Sci Rep. 2022 Mar 2;12:3432. doi: 10.1038/s41598-022-07484-x (PMC8891288; doi:10.1038/s41598-022-07484-x)
Supplement: Supplementary file 1 — Supplementary Information 1. [file 41598_2022_7484_MOESM1_ESM.pdf]

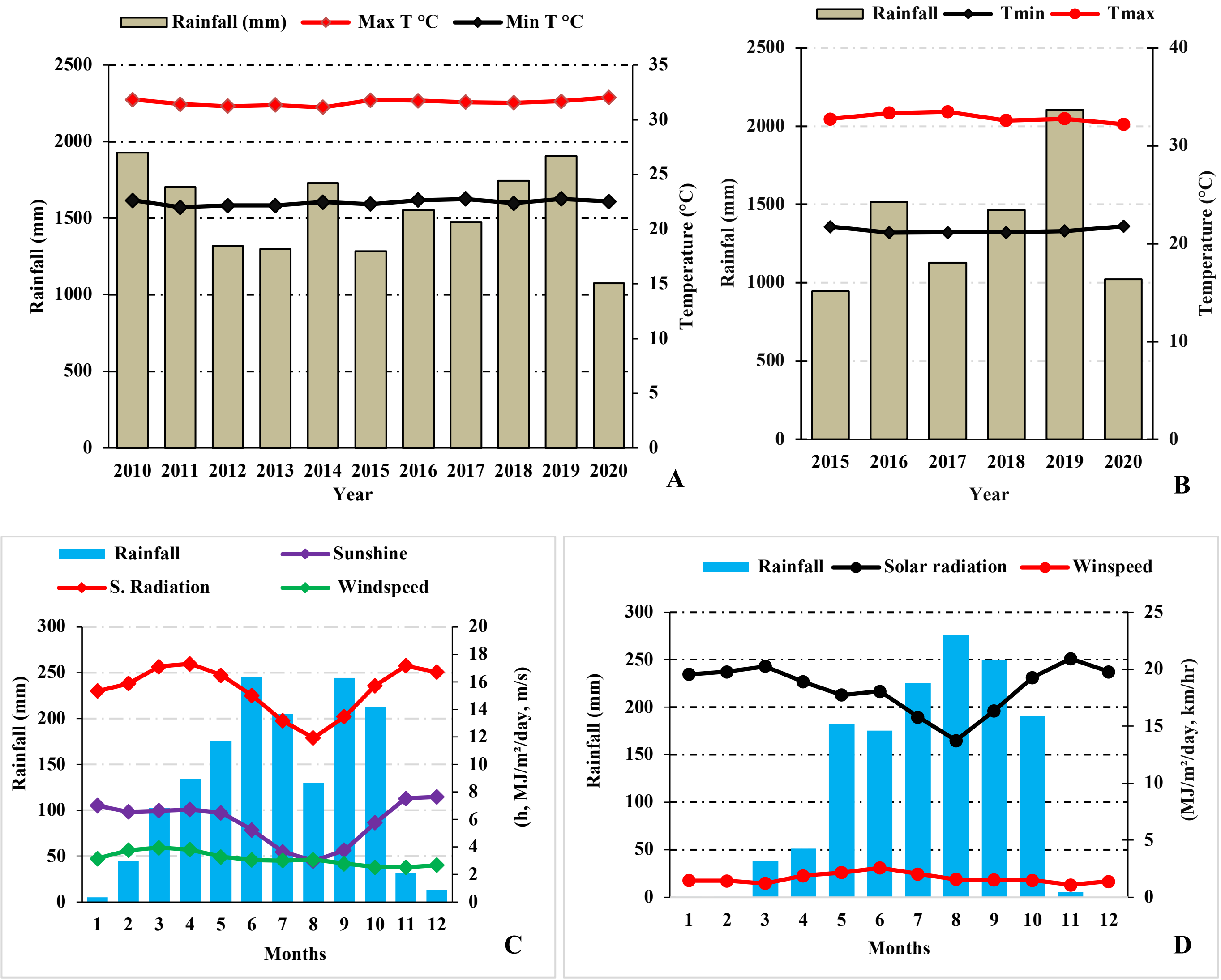

FIGURE S1. Weather data at IITA breeding stations: **(A)** annual rainfalls and temperatures at Ibadan (2010-2020), **(B)** annual rainfalls and temperatures at Abuja (2015-2020), **(C)** mean monthly rainfall, sunshine, solar radiation, and windspeed at Ibadan (2010-2020), **(D)** mean monthly rainfall, solar radiation, and windspeed at Abuja (2015-2020). Months: 1 corresponds to January and 12 to December.

TABLE S1. Most popular *D. rotundata* cross-combinations and their average crossability rates (ACR) at IITA, 2010-2020

Pedigree	Female	Male	Flowers pollinated	ACR (%)
TDr2826A×TDr1500096	TDr2826A	TDr1500096	1005	58.53[a]
TDr04219×TDr9902789	TDr04219	TDr9902789	2947	43.39[a]
TDr9700917×TDr951932	TDr9700917	TDr951932	3255	43.32[a]
TDr1615003×TDr1613907	TDr1615003	TDr1613907	1341	40.47[a]
TDr1401220×TDr1691044	TDr1401220	TDr1691044	1034	39.73[a]
TDr04219×TDr9501932	TDr04219	TDr9501932	4238	38.81[a]
TDr04219×TDr9902607	TDr04219	TDr9902607	5913	38.78[a]
TDr2826A×TDr2946A	TDr2826A	TDr2946A	2059	38.73[a]
TDr1679070×TDr1689247	TDr1679070	TDr1689247	1307	38.00[a]
TDr1669010×TDr1621013	TDr1669010	TDr1621013	1140	37.62[a]
TDr04219×TDr9700777	TDr04219	TDr9700777	2230	36.86[a]
TDrAkunchi×TDr1100034	TDrAkunchi	TDr1100034	1542	35.53[a]
TDr1669010×TDr1669009	TDr1669010	TDr1669009	1019	35.31[a]
TDr1100873×TDr9501932	TDr1100873	TDr9501932	1491	35.29[a]
TDr9700632×TDr9902607	TDr9700632	TDr9902607	3379	34.22[a]
TDr9700632×TDr9501932	TDr9700632	TDr9501932	1521	33.66[a]
TDr1100873×TDrFaketsa	TDr1100873	TDrFaketsa	1301	33.45[a]
TDr9700917×TDr1101039	TDr9700917	TDr1101039	1021	32.01[a]
TDr9700917×TDr9902607	TDr9700917	TDr9902607	11027	31.97[a]
TDrPanpas×TDr9700940	TDrPanpas	TDr9700940	1582	30.88[a]
TDr9700917×TDr8902677	TDr9700917	TDr8902677	2377	30.69[a]
TDr9700917×TDr9501932	TDr9700917	TDr9501932	4552	30.07[a]
TDr9700917×TDrLeusi	TDr9700917	TDrLeusi	1208	28.94[a]
TDrPepa×TDr9501932	TDrPepa	TDr9501932	1091	27.87[a]
TDr9700917×TDr9902789	TDr9700917	TDr9902789	4991	27.77[a]
TDrAkunchi×TDr9501932	TDrAkunchi	TDr9501932	1163	27.35[a]
TDr04219×TDr0615	TDr04219	TDr0615	1283	25.94[a]
TDr1615003×TDr1613401	TDr1615003	TDr1613401	1321	25.33[a]
TDr9519156×TDr9501932	TDr9519156	TDr9501932	1894	24.88[a]
TDr2826A×TDr0800068	TDr2826A	TDr0800068	1004	24.53[a]
TDr1400359×TDr1691044	TDr1400359	TDr1691044	1123	23.92[a]
TDr1615003×TDr1613701	TDr1615003	TDr1613701	2144	21.20[b]
Meccakusa×TDr0000362	Meccakusa	TDr0000362	2010	21.02[b]
TDr9700917×TDr04219m	TDr9700917	TDr04219m	1029	19.92[b]
TDr0500491×TDr0800068	TDr0500491	TDr0800068	1922	19.65[b]
Meccakusa×TDr9501932	Meccakusa	TDr9501932	2949	19.07[b]
TDr0500491×TDr9501932	TDr0500491	TDr9501932	1220	18.77[b]
TDrOjuIyawo×TDr9501932	TDrOjuIyawo	TDr9501932	2329	18.57[b]
TDr9518544×TDr0700217	TDr9518544	TDr0700217	1277	18.53[b]
Meccakusa×TDr9902607	Meccakusa	TDr9902607	2397	18.05[b]
TDr9700917×TDr9700777	TDr9700917	TDr9700777	7792	17.94[b]
TDr9700205×TDr9902607	TDr9700205	TDr9902607	1429	16.71[b]
TDr9518544×TDr0900058	TDr9518544	TDr0900058	2208	16.57[b]
TDr9600629×TDr9501932	TDr9600629	TDr9501932	1327	15.28[b]
TDrNduu×TDr1100114	TDrNduu	TDr1100114	1807	14.78[b]
TDr9700793×TDr9902607	TDr9700793	TDr9902607	1288	14.38[b]
TDr9700793×TDr9501932	TDr9700793	TDr9501932	2403	13.24[b]
TDrNduu×TDr9902789	TDrNduu	TDr9902789	1548	12.39[b]
TDr9700917×TDr9902626	TDr9700917	TDr9902626	4534	12.00[b]
TDrHembakwase×TDr04219	TDrHembakwase	TDr04219	1330	11.35[b]
TDr9700917×TDr0800068	TDr9700917	TDr0800068	2385	11.15[b]
TDr04219×TDr04219	TDr04219	TDr04219	1916	11.02[b]
TDrNduu×TDr0615	TDrNduu	TDr0615	1013	10.88[b]
TDr9518544×TDr0800068	TDr9518544	TDr0800068	1223	9.22[b]
Meccakusa×TDr1000912	Meccakusa	TDr1000912	1262	8.07[b]
TDr9700793×TDr9902789	TDr9700793	TDr9902789	2175	7.89[b]
TDrNduu×TDr1100497	TDrNduu	TDr1100497	1473	7.68[b]
TDr2826A×TDr1499A	TDr2826A	TDr1499A	1148	6.54[b]
TDr9700917×TDrEbute	TDr9700917	TDrEbute	1490	5.75[b]
Meccakusa×TDr0800068	Meccakusa	TDr0800068	2455	5.73[b]
TDrHembakwase×TDr1100461	TDrHembakwase	TDr1100461	1672	5.11[b]
TDr9518544×TDr0500491	TDr9518544	TDr0500491	1198	3.73[b]
TDr9700917×TDrPouna	TDr9700917	TDrPouna	2624	3.13[b]
TDr1400359×TDr1401785	TDr1400359	TDr1401785	1218	2.39[b]

Meccakusa×TDr04219	Meccakusa	TDr04219	2271	1.30[b]
TDr9700917×TDr1499A	TDr9700917	TDr1499A	1357	0.50[b]

a, b = means above and below the overall *D. rotundata* crossability rate (23.4%), respectively. ACR = average crossability rate

TABLE S2. Most popular *D. rotundata* breeding lines at IITA and the cross-compatibility indices, 2010-2020

SN	Genotypes	Sex	Crosses	Combinations	ACR (%)	SPE	Best match	Best CR (%)	PHC (%)	Ploidy status	Trait of interest	Type of variety
1	TDr9700917	Female	45775	82	17.31	6.89	TDr951932	43.32	34.15	2x	Boiling quality	Breeding line
2	TDr04219	Female	42442	67	18.39	3.56	TDr9600582	53.65	41.93	2x	YMV resistance	Landrace
3	TDr9501932	Male	36421	72	23.85	8.23	TDr9519158	52.95	43.06	2x	Boiling quality	Breeding line
4	TDrMeccakusa	Female	24818	46	7.76	3.05	TDr0000362	21.02	13.04	2x	Boiling and pounding quality	Landrace
5	TDr9902607	Male	20714	52	25.31	10.69	TDr04219	48.24	46.15	2x	Boiling quality	Breeding line
6	TDr9902789	Male	15435	31	22.65	8.10	TDr04219	43.39	35.48	2x	Boiling quality	Breeding line
7	TDr9518544	Female	14981	49	13.76	3.81	TDr1500031	30.07	30.61	2x	General cooking quality	Breeding line
8	TDr9700793	Female	13240	51	8.22	3.77	TDr9902607	14.38	0.00	2x	Early maturity	Breeding line
9	TDr9700777	Male	12769	33	20.17	2.59	TDr04219	36.86	42.42	2x	Early maturity	Breeding line
10	TDrNduu	Female	12384	33	15.13	3.41	TDr8902677	27.49	9.10	2x	Nutrient use efficiency	Landrace
11	TDrAkunchi	Female	11230	37	26.00	6.24	TDr8902677	48.15	51.35	2x	Yield and Boiling quality	Landrace
12	TDr0800068	Male	10684	18	13.96	4.84	TDr2826A	24.53	38.89	2x	Boiling and pounding quality	Breeding line
13	TDrPanpas	Female	10624	34	23.94	8.44	TDr9700940	30.88	14.71	2x	Boiling and pounding quality	Landrace
14	TDr2826A	Female	9841	24	29.79	8.94	TDr1500096	58.53	50.00	2x	General cooking quality	Landrace
15	TDr9700632	Female	9750	39	26.35	11.21	TDr9902607	34.22	48.72	2x	Tuber oxidation	Breeding line
16	TDr9902626	Male	8989	19	14.30	5.53	TDr04219	22.70	5.26	2x	General cooking quality	Breeding line
17	TDr8902677	Male	7813	27	23.97	10.65	TDrAkunchi	48.15	48.15	2x	Early maturity	Breeding line
18	TDr9700205	Female	6859	41	12.92	3.52	TDr9501932	21.15	0.00	2x	General cooking quality	Breeding line
19	TDr9519156	Female	6728	19	21.36	4.68	TDrFaketsa	29.89	47.37	2x	Early maturity	Breeding line
20	TDrFaketsa	Male	6193	46	23.65	5.22	TDr1100873	33.45	36.96	2x	General cooking quality	Landrace
21	TDrHembakwase	Female	5841	49	12.33	1.87	TDrGBANGU	13.67	0.00	2x	General cooking quality	Landrace
22	TDr0000362	Male	5785	18	21.89	8.47	TDr0300139	41.89	27.78	2x	Multiple tubering	Breeding line
23	TDr0900058	Male	5518	15	18.44	0.75	TDrAkunchi	33.14	40.00	2x	High Dry matter	Breeding line
24	TDr1100873	Female	5007	18	31.85	5.05	TDr9501932	35.29	44.44	2x	Tuber flesh colour and dry matter	Breeding line
25	TDrOjuIyawo	Female	4686	15	7.16	6.60	TDr9501932	18.57	0.00	2x	General cooking quality	Landrace
26	TDrUfenyi	Female	4392	25	15.91	5.82	TDr9501932	22.10	24.00	2x	High dry matter	Landrace
27	TDrAlumaco	Male	4387	14	26.59	5.18	TDr1100873	31.62	64.29	2x	General cooking quality	Landrace
28	TDr1400359	Female	4344	19	17.69	0.00	TDr1613902	29.19	36.84	2x	General cooking quality	Breeding line
29	TDrEbute	Male	4323	8	6.75	3.72	TDr9518544	10.70	0.00	2x	Yield and boiling quality	Landrace
30	TDr9902562	Male	4301	13	21.24	5.04	TDr04219	33.57	53.85	2x	Nutrient use efficiency	Breeding line

ACR=average crossability rate, SPE=seed production efficiency, PHC=percentage high crossability, CR=crossability rate

TABLE S3. Most popular *D. alata* cross-combinations and their average crossability rates at IITA, 2010-2020

Pedigree	Female	Male	Flowers pollinated	ACR (%)
TDa0500015×TDa9801174	TDa0500015	TDa9801174	1168	57.61[a]
TDa0500015×TDa9900048	TDa0500015	TDa9900048	1150	54.25[a]
TDa0500015×TDa0200012	TDa0500015	TDa0200012	2007	46.95[a]
TDa0000005×TDa0100039	TDa0000005	TDa0100039	2363	46.13[a]
TDa0000005×TDa0200012	TDa0000005	TDa0200012	2794	41.50[a]
TDa9900240×TDa0200012	TDa9900240	TDa0200012	2324	36.08[a]
TDa9801166×TDa0200012	TDa9801166	TDa0200012	2639	34.42[a]
TDa9900240×TDa9801174	TDa9900240	TDa9801174	1236	31.10[b]
TDa0500015×TDa8500250	TDa0500015	TDa8500250	1899	25.55[b]
TDa9900240×TDa8500250	TDa9900240	TDa8500250	1602	23.72[b]
TDa0900376×TDa0200012	TDa0900376	TDa0200012	1481	22.66[b]
TDa0000005×TDa950310	TDa0000005	TDa950310	1811	16.12[b]
TDa0900217×TDa0100039	TDa0900217	TDa0100039	1046	13.22[b]
TDa0000005×TDa950310	TDa0000005	TDa950310	1134	12.17[b]
TDa0800007×TDa0200012	TDa0800007	TDa0200012	1128	6.06[b]
TDa0900217×TDa1100010	TDa0900217	TDa1100010	2144	4.44[b]
TDa161001×TDa160810	TDa161001	TDa160810	1050	3.50[b]
TDa0000005×TDb3690	TDa0000005	TDb3690	1777	3.27[b]
TDa0900217×TDa0900026	TDa0900217	TDa0900026	1026	2.12[b]
TDa0900217×TDa9801174	TDa0900217	TDa9801174	1016	1.83[b]

a, b = means above and below the overall *D. alata* crossability rate (31.7%), respectively. ACR=average crossability rate

TABLE S4. Most popular *D. alata* breeding lines at IITA and cross-compatibility indices, 2010-2020

SN	Genotype	Sex	Crosses	Combinations	ACR (%)	SPE	Best match	Best CR (%)	PHC (%)	Ploidy status	Trait of interest	Type of variety
1	TDa0000005	Female	21995	71	26.58	5.36	TDa1401132	76.19	29.58	-	Boiling quality and Anthracnose resistance	Breeding line
2	TDa0200012	Male	19300	53	32.71	9.06	TDa1500121	82.71	54.72	-	Yield	Breeding line
3	TDa9900240	Female	11911	51	27.09	6.66	TDa0200012	36.08	39.22	2x	Tuber oxidation and earliness	Breeding line
4	TDa0500015	Female	11706	45	32.31	11.86	TDa0100039	64.39	31.11	-	Anthracnose resistance and boiling quality	Breeding line
5	TDa0900217	Female	9780	32	14.66	3.88	TDa0900351	64.62	43.75	-	Drought tolerance	Breeding line
6	TDa8500250	Male	8953	43	17.34	3.92	TDa9500328	27.00	0.00	-	Anthracnose resistance and earliness	Breeding line
7	TDa0100039	Male	7417	25	37.56	11.63	TDa0500015	64.39	52.00	2x	Anthracnose resistance	Breeding line
8	TDa95310	Male	7131	20	18.97	4.44	TDa9900240	34.38	20.00	-	Tuber shape and flesh colour	Landrace
9	TDa0100081	Female	6385	41	27.10	11.88	TDa0100039	59.48	43.90	2x	General cooking quality	Breeding line
10	TDa9801174	Male	6101	19	0.00	0.00	TDa0500015	57.61	26.32	-	High yield and boiling quality	Breeding line
11	TDa0900376	Female	5266	26	22.71	7.01	TDa0900026	39.43	53.84	-	Anthracnose resistance	Breeding line
12	TDa0900026	Male	4920	16	17.52	4.78	TDa0900376	39.43	25.00	-	Anthracnose resistance	Breeding line
13	TDa9801166	Female	4749	25	28.95	6.71	TDa0200012	34.42	40.00	-	High yield and Nutrient use efficiency	Breeding line
14	TDa9500328	Female	4537	27	20.55	4.85	TDa8701091	34.53	48.15	2x	Drought tolerance and Earliness	Breeding line
15	TDa98150	Male	4320	27	20.96	4.54	TDa0500015	33.78	37.04	-	Boiling quality, Tuber shape and flesh colour	Landrace
16	TDa9900048	Male	3844	23	39.57	13.50	TDa0500015	54.25	60.87	-	General cooking quality and earliness	Breeding line
17	TDa8701091	Male	3753	27	26.57	6.46	TDa9500328	34.53	48.15	2x	Anthracnose resistance	Breeding line
18	TDa1100010	Male	3713	14	5.38	1.63	TDa0500015	11.45	0.00	2x	Tuber flesh colour	Breeding line
19	TDa0000194	Female	3563	26	18.47	5.23	TDa0200012	42.46	15.38	-	Yield, boiling and flour quality	Breeding line
20	TDa1100295	Male	2839	11	19.74	8.67	TDa0100081	31.56	18.18	-	General cooking quality	Breeding line
21	TDa160810	Male	2654	9	7.24	0.00	TDa160303	25.87	0.00	-	Yield and tuber quality	Breeding line
22	TDa1662002	Female	2552	9	9.15	0.00	TDa1662006	15.61	0.00	-	Tuber shape	Breeding line
23	TDa0100004	Male	2482	8	12.18	4.45	TDa0100081	22.51	0.00	2x	General cooking quality	Breeding line
24	TDa1100192	Male	2345	11	6.89	0.98	TDa9900240	13.19	0.00	-	General cooking quality	Breeding line
25	TDa0100041	Female	2164	25	12.83	1.93	TDa0200012	27.27	0.00	-	General cooking quality and earliness	Breeding line

ACR=average crossability rate, SPE=seed production efficiency, PHC=percentage high crossability, CR=crossability rate

TABLE S5. Pollinator effects on *D. alata* and *D. rotundata* average crossability rates (2020)

D. alata		*D. rotundata*	
**Technicians***	**ACR (%)**	**Technicians**	**ACR (%)**
T1	18.22±30.56[ab]	T1	27.91±23.96[c]
T2	8.79±19.39[b]	T2	47.70±21.64[abc]
T3	12.50±25.42[ab]	T3	41.79±22.60[abc]
T4	6.25±19.03[b]	T4	59.24±23.93[ab]
T5	17.70±28.72[ab]	T5	67.37±22.25[a]
T6	30.32±27.08[a]	T6	38.18±24.22[abc]
T7	15.33±27.28[ab]	T7	37.91±29.20[bc]
T8	17.44±29.29[ab]	T8	30.34±29.20[c]
Mean	14.88±26.57 (3492)	Mean	33.07±26.43 (8387)

**Technicians' codes for D. alata are not referring to the same individuals as for D. rotundata. The generally low fruit sets for D. alata are confounded with the effects of poor weather conditions during the experiment period (2020). Figures in parenthesis represent the number of flowers pollinated. ACR = average crossability rate, p = 0.0023 (D. alata) and p < 0.001 (D. rotundata). Means (± standard deviation) with the same letters are not statistically different at the 5% p-value threshold of the Tukey HSD test.*

TABLE S6. Summary ANOVA of weather parameters' effects on *D. alata* and *D. rotundata* average crossability rates at IITA

**Sources of variation**	*D. alata*			*D. rotundata*		
	**MS**	***p*-value**	**Sign**	**MS**	***p*-value**	**Sign**
Rainfall	739.11	0.06071	.	710.42	0.0279	*
Evaporation	51.978	0.6761	ns	94.844	0.4836	ns
Windspeed	259.57	0.3299	ns	232.58	0.2605	ns
Solar radiation	30.72	0.749	ns	350.28	0.1573	ns
Min temperature	562.18	0.1219	ns	594.04	0.05137	.
Max temperature	1066.90	0.008773	**	947.67	0.006039	**
Min relative humidity	1098.91	0.006598	**	207.11	0.2906	ns
Max relative humidity	4.395	0.9042	ns	744.37	0.02303	*
Sunshine	1190.01	0.002351	**	91.966	0.4906	ns
Rainy days	1003.56	0.01431	*	249.44	0.2423	ns

Signif. codes: '***' 0.001 '**' 0.01 '*' 0.05 '.' 0.1 'ns' non-significant, MS : Mean square.

TABLE S7. Crossbred seed germination rates of *Dioscorea* species (2014-2020)

Year	Yam species	Seed germination rate (%)
2014		44.40±31.87^{bc}
	D. alata	56.72±29.58
	D. rotundata	38.00±31.38
2015		36.71±29.36^{c}
	D. alata	61.04±21.78
	D. rotundata	12.38±6.61
2016		45.24±22.85^{bc}
	D. alata	51.77±22.52
	D. rotundata	40.34±22.07
2017		48.59±27.21^{ab}
	D. alata (OP)	42.80±22.20
	D. rotundata (OP)	56.78±31.50
2020		53.84±25.91^{a}
	D. alata (OP)	42.85±23.61
	D. rotundata (OP)	66.59±18.05
	D. rotundata	61.19±25.88
Species means	*D. alata*	55.21±24.89^{ab}
	D. alata (OP)	42.84±23.12^{c}
	D. rotundata	49.42±29.22^{b}
	D. rotundata (OP)	61.40±26.36^{a}
	Inter-specific crosses	43.83±33.79^{bc}
Overall means		49.66±27.08

Yearly and species mean (± standard deviation) with the same letters are not statistically different at 5% p-value threshold of the least significant difference (LSD) test, p = 0.0003 (year) and p < 0.001 (species). OP stands for open-pollinated. Interspecific crossbred seeds are those from cross-combinations in ***Table 2.***

Table S8. *D. alata* and *D. rotundata* cross-combinations with superior average seed germination rates at IITA, 2014-2020

Intraspecific cross-combinations		Open pollination	
Family	Seed germination rate (%)	Female	Seed germination rate (%)
TDr9700632×TDr9902607	73.51	TDr1689	79.00
TDr9700632×TDr9501932	66.24	TDr1683	65.10
TDr9700917×TDr8902677	52.93	TDr1685	62.60
TDr04219×TDr0615	54.98	TDr1684	71.21
TDr9700205×TDr9501932	81.19	TDr1697	80.22
TDr1000459×TDr1401419	81.60	TDr1680	81.85
TDr1100873×TDa1400268	88.00	TDr1617604	78.40
TDr1100873×TDr0900324	86.80	TDr1617709	70.00
TDr1301550×TDr0900070	59.60	TDr1617711	70.80
TDr1401220×TDr9501932	72.40	TDr1617803	61.60
TDr14020266×TDr0900082	49.20	TDr1617908	64.80
TDr2826A×TDr1500096	64.80	TDr1618005	86.80
TDr2826A×TDr1500128	58.00	TDr1618103	83.60
TDr2826A×TDr1500135	80.80	TDr1618507	80.40
TDr3010×TDr1500031	88.00	TDr1618707	67.20
TDr3010×TDr1500042	83.60	TDr1618714	66.80
TDr3010×TDr1500043	58.80	TDr1619010	86.80
TDr3010×TDr1500096	89.20	TDr1620601	85.20
TDr3010×TDr1500100	81.20	TDr1621001	64.00
TDr3010×TDr1500135	90.00	TDr1621101	83.60
TDr9518544×TDr0800756	84.80	TDr1621401	93.20
TDr9518544×TDr0909132	93.20	TDr1621406	87.60
TDr9518544×TDr1500031	90.00	TDr8902665	68.40
TDr9518544×TDr1500043	68.40	***D. alata***	
TDr9518544×TDr1500100	79.20	TDa1655	58.98
TDr9518544×TDr2948A	88.00	TDa1614AB	45.31

TDr9700632×TDr8902677	64.49	TDa19001	76.80
TDr9700917×TDr1500096	66.00	TDa19014	77.60
TDr9700917×TDr1500135	62.40	TDa19015	65.20
TDrAgbanwobe×TDr0900324	71.20	TDa19017	74.00
TDrHEMBAKWASE×TDr2363A	83.60	TDa19028	48.80
TDrpampas×TDr1401785	62.80	TDa19029	86.40
D. alata		TDa19036	61.20
TDa0900217×TDa0900351	85.32	TDa19037	62.00
TDa0000005×TDa0200012	76.20	TDa19047	56.00
TDa0500015×TDa8500250	60.70	TDa19048	58.40
TDa0000005×TDa9801174	66.22	TDa19078	66.40
TDa0000005×TDa0100039	78.13	TDa19080	90.40
TDa9900240×TDa0200012	77.37	TDa19082	49.60
TDa0900217×TDa0200012	60.26	TDa19084	62.80
TDa9900240×TDa9801174	64.93	TDa19089	67.60
TDa0100081×TDa0200012	64.19	TDa19090	68.40
TDa9500328×TDa0200012	57.95	TDa19116	49.60
TDa0100081×TDa0100039	66.42		

TDr= D. rotundata, TDa=D. alata, OP=Open pollinated. These families/females were superior to the overall means for each species as presented in ***Table*** *7. Only samples above 250 seeds sown were considered in determining seed germinations rates.*

Table S9. Traits of interest targeted in interspecific crosses at IITA

**Species**	**Trait of interest**
D. bulbifera	Introgress the ability to produce aerial bulbils for use as alternative planting materials owing to the high cost and supply shortage of seed yam propagules
D. praehensilis	Source of genes for adaptation traits and tolerance to yam mosaic virus as well as improved carotenoid content
D. cayenensis	Source of carotenoids due to its yellow flesh colour
D. dumetorum	Resistance/tolerance to nematodes
D. burkilliana	Bridging and introgress disease tolerance and the ability to produce aerial bulbils for use as alternative planting materials
D. hirtiflora	Bridging and introgress disease tolerance and the ability to produce aerial bulbils for use as alternative planting materials
D. alata	Introduction of genes for high vigour and yield from *D. alata* to *D. rotundata*
